# The Urine in Disease

**Published:** 1884-07

**Authors:** 


THE URINE IN DISEASE.—Accompanying the "Medical World," we have received a chart with the above title, which contains a systematically arranged summary of the different pathological conditions of the urine. It shows at a glance its abnormal ingredients, specific gravity, reaction, etc.; gives the various tests, and notes the therapeutical indications. Veterinarians would find it full of practical suggestions in the study of urinary diseases in animals. The chart is sent gratis to subscribers of the "World" for one year, the price of which s but $1.

